# Supplementary material for: Bacteriophages Biocontrol of Kiwifruit Bacterial Canker Caused by Pseudomonas syringae pv. actinidiae (Psa) in Two Seasons Under Field Conditions
Source: Antibiotics (Basel). 2025 Oct 14;14(10):1023. doi: 10.3390/antibiotics14101023 (PMC12561079; doi:10.3390/antibiotics14101023)
Supplement: Supplementary file 1 [file antibiotics-14-01023-s001.zip › antibiotics-3883684-supplementary.pdf]

Supplementary data

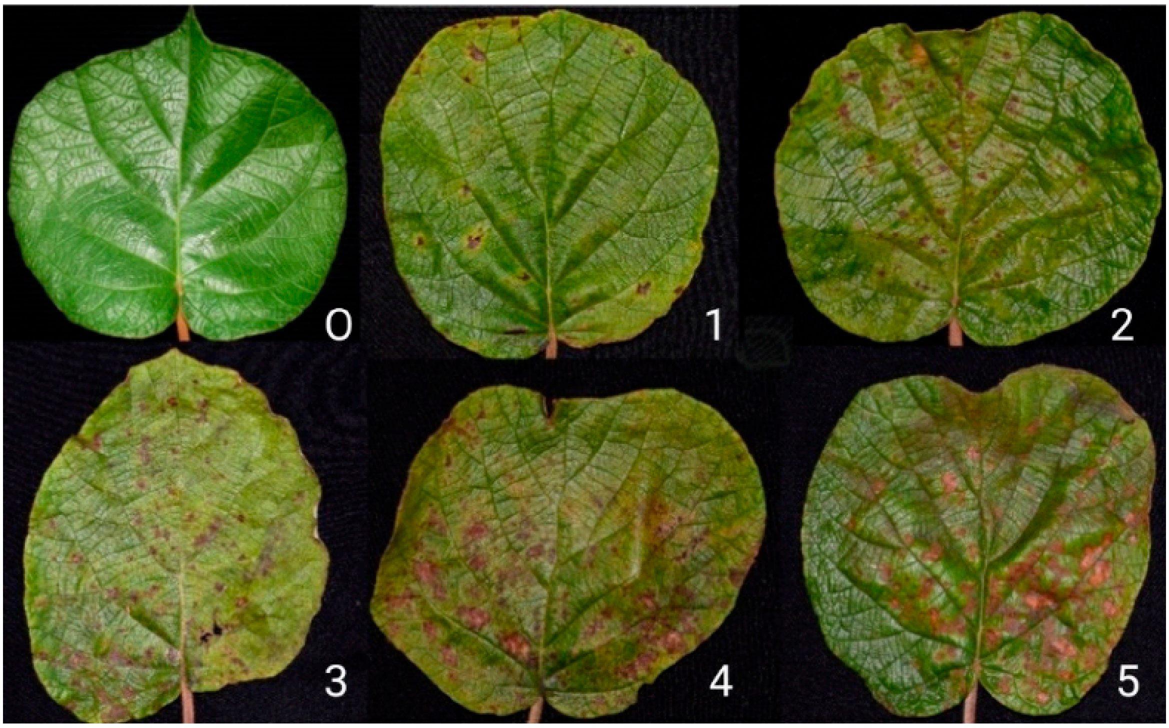

Figure S1. Damage scale for kiwifruit leaves from Flores et al. [42] with the following parameters 0: healthy leaf; 1: 1-4% of leaf area affected; 2: 5-10% of leaf area affected with single spots and few merged spots; 3: 11-30% of leaf area affected with merged spots; 4: 31-49% of leaf area affected⍰merged spots covering veins and increasing in size; 5: >50% of leaf area affected.

Table S1. Pathogenicity test performed with *Pseudomonas syringae*

**Isolate**	**Pseudomonas species/pathovar***	**Pathogenicity test 7 days PI**	**Pathogenicity test 15 days PI**
**FPL7**	*P.s. actinidae*	Positive	Positive
**3C**	*P.s.actinidae*	Positive	Positive
**FPL11**	*P.s. actinidae*	Positive	Positive
**BP2**	*P, s. syringae*	Positive	Positive
**BP4**	*P.s. syringae*	Positive	Positive
**BP10**	*P.s. syringae*	Positive	Positive
**F1**	*P.s. syringae*	Positive	Positive
**BM1**	*P.s. syringae*	Positive	Positive
**BP7**	*P.s. syringae*	Negative	Positive

**BP1**	*P.s. syringae*	Positive	Positive
**AM3**	*P.s. syringae*	Negative	Positive
**BP12**	*P.s. syringae*	Positive	Positive
**BP8**	*P. viridiflava*	Negative	Negative
**Negative control**	Only water	Negative	Negative

* According to the results observed in Table 2
PI Post inoculation
